# Injury incidence and specific injury patterns in app-based bodyweight training (Freeletics): results of an international survey with 3668 participants

**DOI:** 10.1186/s13102-022-00525-y

**Published:** 2022-07-26

**Authors:** G. Hertel, A. Hochrein, C. Suren, P. Minzlaff, I. J. Banke, J. Willers, R. von Eisenhart-Rothe, P. M. Prodinger

**Affiliations:** 1 Klinik und Poliklinik für Orthopädie und Sportorthopädie, Klinikum rechts der Isar, Technische Universität München, Ismaninger Straße 22, 81675 Munich, Germany; 2 OCM Orthopädische Chirurgie München, Munich, Germany; 3 Städtisches Krankenhaus München-Bogenhausen, Orthopädie, Munich, Germany; 4 Krankenhaus Agatharied, Abteilung für Unfallchirurgie und Orthopädie, Hausham, Germany

**Keywords:** Bodyweight, Freeletics, Workout, App, International, Training, Injury patterns

## Abstract

**Background:**

The aim of this study was to analyze incidences and sport-specific injury patterns among users of a bodyweight-based training method instructed by a smartphone app (*Freeletics Bodyweight App*).

**Methods:**

An online questionnaire based on current validated epidemiological observation methods was designed using the statistic website Surveymonkey. Subscribers of the *Freeletics Bodyweight App* were contacted via an online link. Injury incidence, defined as an event leading to a training pause of at least 1 day, was recorded. The type of injury was reported and classified. Furthermore, all participants were asked whether they recognized any positive or negative effects on their subjective health status. The collected data were analyzed using *Surveymonkey* statistic services.

**Results:**

A total of 4365 *Freeletics* users responded to the questionnaire, 3668 completed forms were subject of further investigation. The injury period prevalence reported by users of the *Freeletics App* was 24% in men and 21% in women. The most frequently reported site of injury was the shoulder (29%) and the knee joint (28%), with strains (28.5%) and other muscle injuries (14.4%) being the most frequently reported types of injuries. An injury incidence rate of 4.57 per 1000 h was calculated, with injuries occurring less frequently in experienced users. Most participants reported a distinct positive effect of the app-based training on their health status.

**Conclusion:**

In comparison to other sports activities app-based bodyweight training is associated with a comparably low injury period prevalence. The vast majority of injuries were reported to have resolved within one week.

**Supplementary Information:**

The online version contains supplementary material available at 10.1186/s13102-022-00525-y.

## Background

Functional and free bodyweight training methods have rapidly increased in popularity over the past years. Bodyweight training can promote physical fitness and can be performed independently at home. Training methods are similar to *high intensity functional training* (HIFT), emphasizing varied functional movements such as lifting, pulling, and throwing at relatively high intensity. These programs are designed to promote general physical preparedness [1]. *Extreme conditioning programs* (ECPs), are defined as fitness training regimens relying on aerobic, plyometric, and resistance training exercises, often with high levels of intensity for a short duration of time [2]. Both methods are effective in weight loss [3] and have positive effects on strength, metabolic conditioning performance and body composition [4]. They are components of traditional (not app-based) programs such as CrossFit. The injury rate of CrossFit is lower than, or comparable to other forms of exercise or strength training [5], even though cases of severe injuries have been reported [6, 7].

The *Freeletics Bodyweight App* has more than 51 million registered users in 175 countries [8]. It allows training without additional weights or equipment. Exercise instructions are given by the app, training intensity and composition can be adjusted based on the user’s feedback to reduce the probability of overuse injuries. Thus, this negative side effect already reported in other ECPs can occur [9]. Additionally training with an app offers a direct comparison to other users in an online community, possibly putting pressure on participants to train too intensely. Easy access to the training methods makes them furthermore eligible for primarily less trained persons.

Currently little data is available regarding injury prevalence and incidence in bodyweight-based training methods. Data about injury incidence and specific injury patterns in app-based training methods are lacking [10–12].

The aim of this study was to analyze the incidence of injuries among *Freeletics* users and to evaluate them in respect to location, period prevalence, severity and correlation to training intensity and respective exercises.

## Methods

This work was designed as descriptive epidemiological study. An online survey was built to enable worldwide data collection without spatial or temporal limitations. Standardized development of the questionnaire began with the determination of the research questions, production of the primary version and internal testing for validity, followed by the finalization of the survey. The survey was created on the U.S.-American online platform www.surveymonkey.com. It contains various questions concerning demographics (e.g. participants' age, sex, country of origin), *Freeletics* training experience and intensity (e.g. duration of training, sessions per week, average training hours) and reported injuries within the past 9 months (e.g. type of injury, injured body region, resulting training pause). The full survey (German and an English version) is available as Additional file 1.

The survey was uploaded in its final version (for mobile devices also) on the 8th of December 2017. An HTML link with description was sent via an email newsletter by the company *Freeletics* to 88,000 randomly chosen subscribers, who were active users at the time of the survey. Continuous information on the number of participants and the time required for completion was given. The survey was closed on February 12th, 2018 and the HTML links deactivated.

*Surveymonkey* guarantees data encryption and is certified by EU-U.S. Privacy Shield. Participants’ information was anonymized, an IP-based block was used to ensure every user could only participate once.

Statistical analysis was provided by *Surveymonkey’s* analytical services, further specifications are available on their website [13]. Only fully completed questionnaires were analyzed. A confidence level of 95% (*p* < 0.05) was set. A two-sample t-test was used for comparisons amongst groups [13]. In order to compare injury incidence to other sports activities, the incidence rate per 1000 h exposure was calculated according to the method described by Philips [14].

An injury was defined as any event leading to a training pause of at least one day [14, 15]. We designed the questionnaire defining the severity of injuries based on the study by Clarsen et al. which showed that overuse injuries were formerly frequently undetected [16]. Strains, tendinopathies, abrasions, muscle injuries, bruises and periostitis were defined as minor injuries. Fractures, dislocations, bone marrow edemas and cartilage damage were defined as severe injuries. Prevalence and incidence measurements were described according to Nielsen et al. [17]

## Results

Within 2.5 months, 4365 participants started the survey. Completion rate of the questionnaire was 84%, with 3668 respondents from 63 countries. 2433 respondents completed the German questionnaire (66.3%) and 1235 respondents completed the English version (33.7%).

Both language groups showed the same age distribution. The largest age group was 31–35 years of age (22%), followed by the 26–30 year-olds (20%) and the 36–40 year-olds (18%). Sixty-six percent of the respondents to the German version of the questionnaire were male (*n* = *1599*), 34% (*n* = *834*) female. Eighty-two percent of the respondents to the English version were male (*n* = *1012*), 18% female (*n* = *223*).

Thirteen percent of the participating users had begun their *Freeletics* training 1–3 months before completing the survey, 11% had been training for 5–6 months. The vast majority was experienced and had started at least half a year before the survey. Eighty-eight percent of all participants trained alone. Ten percent alternated between training in a group and training alone, only 2% of the participants exclusively trained in groups.

Seventy percent of the participants performed at least three training sessions per week (Table 1).

**Table 1:** Training sessions and duration

Number of workouts $\left[\mathrm{per week}\right]$	Percent $\left[\%\right]$	Time of workout $\left[\mathrm{min}\right]$	Percent $\left[\%\right]$
$\le$1	12	$\le$ 15	5
2	18	16–30	37
3	40	31–45	45
4	22	45–60	11
$\ge$ 5	8	$\ge$ 60	2

Sixty-three percent of the participants were regularly involved in other sports apart from *Freeletics* training. The most common activities were jogging, cycling, bodybuilding and swimming. Six percent also used other fitness apps (Table 2).

**Table 2:** Sports activity apart from Freeletics and resting time

Activities in addition to Freeletics $\left[\mathrm{per week}\right]$	Percent $\left[\%\right]$	Resting time per week $\left[\mathrm{day}\right]$	Percent $\left[\%\right]$
< 1	5	< 1	2
1	24	1	14
2	33	2–3	68
3	22	4	13
> 3	16	> 4	3

### Injury frequency

Twenty-four percent of the participating users suffered at least one injury during *Freeletics* training in the last 9 months preceding the survey. There was no significant difference of injury prevalence in different countries (*p* < 0.05). Age, sex and BMI had no significant influence on the injury prevalence, nor had the amount of daily physical labor (*p* < 0.05).

Participants recorded a combined total of 250,803 training hours during the 9 months recording period. The injury incidence rate was calculated to be 4.57 per 1000 h, from 1147 reported injuries. A higher incidence rate was reported in participants subjectively perceiving the training as more demanding than in participants perceiving it as easy.

**Fig. 1:**
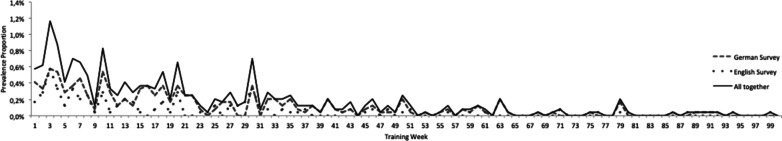
Injury prevalence proportion during the respective training weeks

There was a negative correlation between the incidence of injuries reported and the number of training weeks (Fig. 1).

Fifty-two percent of the respondents reported one injury; 29% had two injuries, 11% three injuries and 7% reported four or more injuries.

Sixty-three percent of the reported injuries were self-diagnosed, 25% were diagnosed by a physician and 12% by a physiotherapist.

**Fig. 2:**
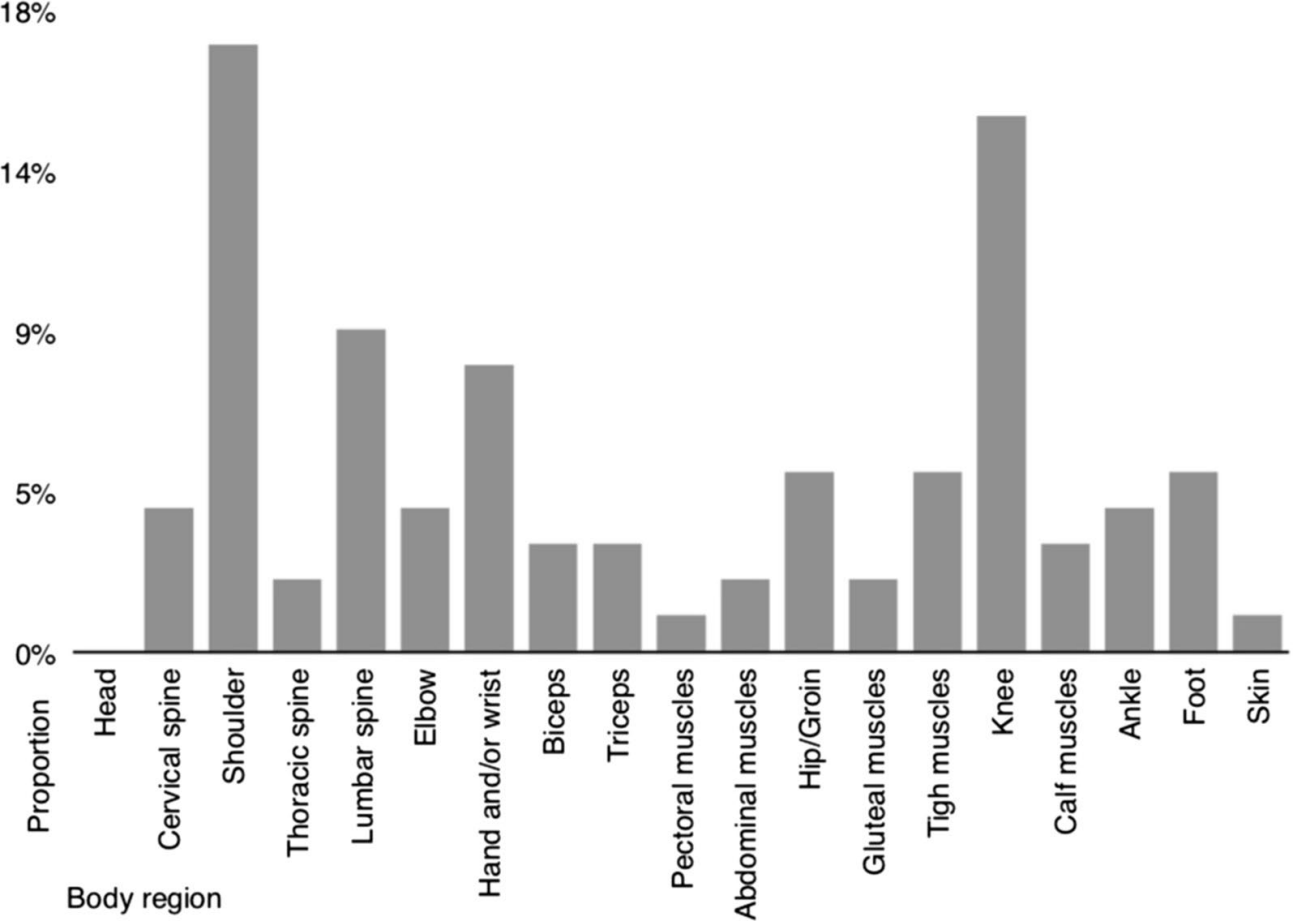
Injury prevalence proportion in respect to body region

Lower limb injuries were the most commonly reported ones (41%), followed by the upper limb (39%), 17% affected the spine and 3% other locations (e.g. head, abdomen). Detailed information for all body regions is given in Fig. 2.

**Fig. 3:**
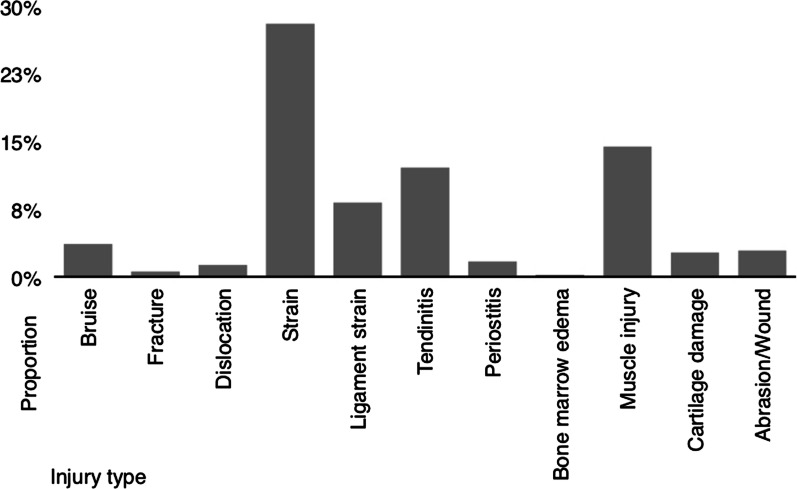
Injury prevalence proportion in respect to injury type

Regarding the types of injuries reported, less severe injuries such as strains were most common (28.5%). Furthermore, muscle injuries (14.4%) and insertion tendinopathies (12.1%), followed by ligament sprains (8.2%) occurred frequently. More severe injuries such as fractures, joint dislocations or cartilage lesions were rarely reported (Fig. 3).

### Injury patterns of different exercises (Table *3**)*

**Table 3:** Injury proportion and location in respect to different exercises

Exercise	Pull up	Muscle up	Push up	Handstand push up	Burpee	Burpee frogs	Froggers	Squat	Pistol	High jump	Climber	Fast sprint	Jumping jack	Sit up	Lunges	Jack knives	Stand ups	Others	Total
Total	124	12	156	14	254	22	4	100	12	49	28	30	27	49	49	3	23	191	1147
$\left[\%\right]$	10.8	1	13.6	1.2	22.1	1.9	0.3	8.7	1	4.3	2.4	2.6	2.4	4,3	4.3	0.3	2	16.7	100
Upper limb $\left[\%\right]$	80	75	87	79	41	27	0	0	8	0	4	0	7	6	0	0	26	34	39
Lower limb $\left[\%\right]$	2	0	3	7	31	55	75	87	92	78	82	93	81	14	94	33	57	48	41
Spine $\left[\%\right]$	14	17	8	7	26	18	25	12	0	22	14	7	11	39	2	33	17	16	17
Others $\left[\%\right]$	5	8	3	7	2	0	0	1	0	0	0	0	0	41	4	33	0	2	4

The greatest proportion of injuries occurred with *Burpees* (22.1%), the second greatest proportion with *Push-ups* (13.6%). As shown in Table 3, upper limb injuries were most commonly reported to occur during *Push-ups, Pull-ups, Handstand Push-ups* or *Muscle-ups*. Injuries of the lower limb were mostly reported with *Lunges*, *Fast-sprints*, *Pistols, Squats, Climbers, Jumping Jacks, High-jumps* or *Froggers*. Mixed exercises (*Burpees, Burpee-frogs, Climbers, Jumping Jacks*) showed mixed injury patterns. *Sit-ups* and *Jack-Knives* mainly showed injuries of the abdominal muscles, spine and also skin abrasions (Table 3).

### Recovery and treatment

Fifty-seven percent of the injured participants recovered within one week (8% one day, 35% had to pause for 2–5 days, 14% 6–7 days) and another 14% recovered within 8–14 days. Ten percent of the participants reported a training pause of 15–30 days, 15% had to refrain from training for more than 30 days due to injury.

Forty-four percent of all injuries could be treated by immobilization, training pause or training adaptation. Twelve percent were treated by a physiotherapist. Eleven percent of injuries received no treatment. For 11% of the reported injuries pain medication was used.

### Injury risk reduction (subjective participant valuation)

Thirty-five percent of respondents believed that injuries could have been prevented by a better training technique. A better warm-up (28%), slower training (19%), less repeats or reduced training volume (12%) were also factors thought to potentially reduce the risk of an injury.

### Physical and psychosocial benefits of the Freeletics training

Ninety-two percent of respondents reported feeling "better" or "much better" after using the *Freeletics App*. Eight percent felt about the same and only 1% felt slightly worse.

Eighty-eight percent of respondents reported that their sports performance had improved with the *Freeletics Bodyweight* training. Additionally, 28% of respondents reported that new friends and social contacts had been established using the app.

## Discussion

The most important finding of this study is an injury incidence rate of 4.57 injuries per 1000 h exposure for users of the *Freeletics Bodyweight App*. Minor strains, muscle injuries and insertional tendinopathies were the most common injuries. The number of injuries decreased with time, suggesting that early injuries, possibly caused by initially unfamiliar excess loading or incorrect technique, could be reduced during the ongoing course.

*Freeletics Bodyweight* is a training method performed using only bodyweight, instructed by a smartphone app. Training techniques like this are becoming increasingly popular around the globe, especially during times of public restrictions such as the recent COVID-19 pandemic. More than 3600 respondents from 63 countries underlines the popularity of the *Freeletics Bodyweight App*.

The *Freeletics Bodyweight App* is based on short high intensity workouts that address the main components of physical fitness: strength, stamina and muscular endurance. Workouts last 15–45 min on average. This training technique can be classified as ECP, defined as high volume workouts with a timed maximal number of repetitions and short rest periods in between. So far, increased injury rates have been associated with ECPs [9]. CrossFit, another training style classified as ECP, has recently been quantitively and qualitatively evaluated concerning injuries. Here, an absolute injury prevalence of 19% was reported [18]. In contrast to most ECPs, *Freeletics* utilizes the users’ own body weight without additional weights, which hypothetically should reduce the injury risk. Still, we calculated an injury prevalence of 24% in our study. Compared to the CrossFit study by Weisenthal et al. [18], we used a stricter definition for an injury, in order to also detect minor overload injuries [15]. In our study, an injury was defined as “*any event within the past 9 months leading to a training pause of at least one day*”. Weisenthal et al. considered an injury an event leading to a pause of more than a week or complaints severe enough to consult a physician. By those standards our calculated injury prevalence would have been 10.3%, which is lower and would support the hypothesis that omission of weights reduces the injury risk. Other non-contact sports without additional equipment, such as running/jogging, in this aspect comparable to *Freeletics Bodyweight* training, show inconsistent injury prevalence between 19.4% and 79.3% in the preexisting literature [19]. 

Investigating injuries in gymnasts, also performing exercises with their own bodyweight, Kolt et al. showed injury incidence rates between 2.63 (elite athletes) and 4.11 (sub-elite) per 1000 h exposure [20]. Those results are comparable to our findings. Similar to our study, the authors were able to show that the injury risk decreases with the exercise frequency and the participants’ experience.

In contrast, contact sports such as rugby (18.2–824.7 per 1000 h) or soccer (0.5–44 per 1000 h) show substantially higher injury incidence rates [21, 22]. Training in fitness centers is reported to have an incidence rate of 7.83 injuries per 1000 h, which is more than our results for free bodyweight training [23]. The injury incidence rates in CrossFit training vary from 2.1 to 3.1 per 1000 h and appear to be lower than in our study [24, 25]. However, the number of participants and the definition of injury is inconsistent in these studies. That data lack comparability [14]. In summary the injury incidence rate among *Freeletics* athletes, evaluated in our study, seems comparable and in line with other fitness/training programs and non-contact sports.

Furthermore, our data shows that the number of injuries decreases with time, suggesting that early injuries possibly caused by initially unfamiliar excess loading or incorrect techniques could be reduced during the duration of the training program. This supports the athletes’ subjective belief that injury risk could be reduced by a better training technique, slower training or reduced training volume.

Although there was a negative trend in the rate of injuries with training weeks, there was an increase of injuries reported in training weeks 50, 63 and 79. This corresponds to so-called *hell weeks*, an intensification of the training plan by the app. Those results support the theory that a high training volume can lead to exhaustion as well as to poorer movement execution and thus increases the risk for injuries [9]. Concordantly, Weisenthal et al. suggested an improvement of motion patterns in order to prevent injuries [18].

The most common injuries were minor strains, muscle injuries and insertional tendinopathies. Independently of the respective injury types, most participants only had to pause training briefly. Similar results have been shown for CrossFit [18]. Our results agree with the preexisting literature, even though injury severity definitions vary. Furthermore, most classification systems would underestimate the prevalence of overuse injuries (for example the quantitative injury severity definition established by Sandelin et al. [26]) which we would have expected to be the most prevalent injuries in our study population. Thus, we designed our questionnaire based on the study by Clarsen et al. [16] and qualitatively defined minor and severe injuries according to van Mechelen [27].

Today, injury prevention programs are essential for many athletic sports [29, 30]. In order to develop a prevention program or to improve the workouts, it is of utmost importance to analyze injury prevalence, incidence and patterns as well as influencing factors that are specifically linked to the respective sport [15, 31, 32]. Especially in an app-based training method such as *Freeletics Bodyweight*, in which exercises, intensity and warm up programs are determined by an app-based algorithm, injury patterns must be distinguished. By doing so, attempts to reduce the injury risk by differently combining exercises or modifying the intensity in the training algorithm are feasible. Furthermore, expansion of group training with support and guidance for less experienced athletes could decrease the injury rate. Weisenthal et al. were able to show in their CrossFit study that the presence of a trainer could improve training performance and thus reduce the risk of injury [18]. In our study 88% of participants claimed to always train alone and only 12% at least alternately in a group, obviously leaving some potential to increase this percentage. Due to the relatively small number of participants attending group trainings in our survey, we were not able to verify this effect in our data.

Sports and regular training lead to a better physical and mental wellbeing in humans [33–35]. Concordantly, 92% of the participants in our study reported to feel a lot better in respect to their subjective feeling of health through *Freeletics Bodyweight* training. Eighty-eight percent of participants stated that *Freeletics Bodyweight* training had improved their performance in other sports activities. This shows that *Freeletics* is suitable as complementary training to other sports and that this whole-body training can help to improve overall performance.

This study has some limitations. The retrospective design of the survey is generally prone to recall bias. Gabbe et al. [28] stated that 80% of athletes could retrospectively remember the exact number of injuries and injured body regions over 12 months. The recall bias of our study evaluating only 9 months should be acceptable in this context. Additionally, the questionnaire was provided online, so participants had to complete it without support. Sixty-three percent of participants diagnosed their injury themselves. There might be a certain bias in the understanding/definition of injuries.

## Conclusion

The present study is the first world-wide survey to analyze injury prevalence, incidence and injury patterns in *Freeletics Bodyweight* training, representing an app-based training method with 51 million registered users in 175 countries. The feedback to our survey and the large absolute number of responses validates the interest in the topic. We calculated an injury incidence rate of 4.57 injuries per 1000 h exposure. Injury prevalence and incidence rates found in our study were comparable to those in other athletic and non-contact sports, even though we chose a very strict injury definition. Results from our study can serve as baseline knowledge for future prospective trials.

## Supplementary Information

**Additional file 1.** Freeletics Medical Survey (English Version).

## Data Availability

Data are available upon request.

## Abbreviations

HIFT: High intensity functional training
ECPs: Extreme conditioning programs
HTML: Hypertext markup language
