# Supplementary material for: Injury incidence and specific injury patterns in app-based bodyweight training (Freeletics): results of an international survey with 3668 participants
Source: BMC Sports Sci Med Rehabil. 2022 Jul 26;14:145. doi: 10.1186/s13102-022-00525-y (PMC9321304; doi:10.1186/s13102-022-00525-y)
Supplement: Supplementary file 1 — Additional file 1. Freeletics Medical Survey (English Version). [file 13102_2022_525_MOESM1_ESM.pdf]

de.surveymonkey.com

Freeletics Medical Survey English

Athlete,

our team from the orthopedic and sports orthopedic clinic of the Clinic Rechts der Isar Technical University of Munich wants to help you to prevent injuries and to work preventively.
We need your help!

We are investigating the injuries that may have occured during a Freeletics workout in **the last 9 month**

(Please only refer to Freeletics Bodyweight do not take on Freeletics Running or Freeletics Gym!)

Please help us by filling out the questionnaire.

Please also participate in the survey if you have **not** sustained any injuries.

The questionnaire will take about 5-10 min, as long as an Ares workout :) !

Thanks for your support!

* 1. What country are you currently living in?

- USA
- England
- Australia
- Other country (please specify)

de.surveymonkey.com

* 2. How old are you?

* 3. Are you male or female?

- male
- female

* 4. What is your **b**ody **m**ass **i**ndex? Weight in kilograms / (body size in meters) ^{2}

(Example: Size 1.80m, Weight: 80kg -> 80 / (1.8x1.8) = 24.69 BMI)

- < 16.0
- 16.0 - < 17.0
- 17.0 - < 18.5
- 18.5 - < 25.0
- 25.0 - < 30.0
- 30.0 - < 35.0
- 35.0 - < 40.0
- > 40.0

Weiter

de.surveymonkey.com

* 5. How physically demanding is your job?

- ○ Mainly sitting activity
- ○ Walking and sitting without physical labor
- ○ Walking, sitting, carrying/lifting
- ○ Demanding physical labor

* 6. Smoker

- ○ Yes
- ○ No

* 7. Do you suffer from other medical conditions?

- ☐ No
- ☐ High blood pressure
- ☐ Asthma
- ☐ Gastrointestinal disorders
- ☐ Diabetes mellitus
- ☐ Psychological disorders
- ☐ Other

de.surveymonkey.com

* 8. When did you start Freeletics?

- ◯ 1-3 months
- ◯ 5-6 months
- ◯ 7-12 months
- ◯ 13-18 months
- ◯ 19-24 months
- ◯ 25-30 months
- ◯ more than 30 months

Zurück Weiter

Powered by
SurveyMonkey
Es ist ganz einfach, eine Umfrage zu erstellen.

de.surveymonkey.com

Freeletics Medical Survey English

* 9. How do you mainly train?

- ◯ In a group
- ◯ Alone
- ◯ Alternating in the group and alone

* 10. Average number of "Coach Days" per week during the last 9 month?

- ◯ < 1
- ◯ 1
- ◯ 2
- ◯ 3
- ◯ 4
- ◯ 5
- ◯ 6
- ◯ 7
- ◯ > 7

de.surveymonkey.com

* 11. How many training minutes **per week** did you complete on average during the last 9 month?

- ◯ < 15min
- ◯ 15-30 min
- ◯ 30-45 min
- ◯ 60 - 90 min
- ◯ 90 - 120 min
- ◯ 120 - 160 min
- ◯ 160- 190 min
- ◯ 190 - 240 min
- ◯ > 240 min

de.surveymonkey.com

* 12. Current training level in the App?

- ◯ 1-5
- ◯ 6-10
- ◯ 11-15
- ◯ 16-20
- ◯ 21-25
- ◯ 26-30
- ◯ 31-35
- ◯ 36-40
- ◯ 40-50
- ◯ 50-60
- ◯ >60

Zurück | Weiter

Powered by
SurveyMonkey
Es ist ganz einfach, eine Umfrage zu erstellen.

de.surveymonkey.com

Freeletics Medical Survey English

* 13. Average time per "Coach day"?

- ◯ 0-15 min
- ◯ 16-30 min
- ◯ 31-45 min
- ◯ 46-60 min
- ◯ 61-75 min
- ◯ 76-90 min
- ◯ > 90 min

* 14. Do you do other sports besides Freeletics regularly?

- ◯ Yes
- ◯ No

Zurück Weiter

de.surveymonkey.com

15. Which and what level?

	Level
Bodybuilding	
Running	
Soccer	
Cycling	✓<br>Beginner<br>Recreational athlete<br>Semi Professional<br>Professional
Rowing	
Swimming	
Basketball	
Boxing	
Climbing	
Yoga	
Track and field	
Martial Arts	
Skiing/Snowboarding	
Tennis	
Triathlon	
Volleyball	

16. How often do you do these sports on average **per week** next to Freeletics?

Zurück Weiter

de.surveymonkey.com

Freeletics Medical Survey English

* 17. Average weekly number of recovery days

* 18. What best describes your average warm-up?

- No warm-up
- Full body exercises (running, rowing, rope skipping, etc.)
- Certain body parts
- As suggested by the FL App

* 19. Did you suffer an injury during FL training in the last 9 month?

(Injury definition: any event causing a training pause of at least 1 day)

- Yes
- No

Zurück | Weiter

de.surveymonkey.com

Freeletics Medical Survey English

* 20. How many injuries did you have in the above period?

21. Who diagnosed the injury?

(Fields must only be filled in if something applies!)

- [ ] Self-diagnosis
- [ ] Doctor
- [ ] Physiotherapist
- [ ] Trainer
- [ ] Other

Zurück Weiter

de.surveymonkey.com

Freeletics Medical Survey English

22. What body part was injured?

(Fields must only be filled in if something applies!)

	Type of injury
Head	
Cervical spine	
Shoulder	
Thoracic spine	
Lumbar spine	
Elbow	
Hand and/or wrist	
Biceps	
Triceps	
Pectoral muscles	
Ab muscles	
Hip/groin	

de.surveymonkey.com

Hip/groin	
Gluteal muscles	
Thigh muscles	
Knee	✓
Calf muscles	
Ankle	
Foot	
Skin	

Dropdown options:

- bruise
- fracture
- dislocation
- strain
- ligament strain
- tendinitis
- Periostitis
- Bone marrow
- muscle injury
- cartilage damage
- Abrasion / wound
- Something else

Other

de.surveymonkey.com

23. During which exercise did this injury occur? Did you warm up prior to this injury?

(Fields must only be filled in if something applies!)

	During which exercise ?	Did you warm up prior to this injury?	In which Coach week?
Head			
Cervical spine			
Shoulder			
Thoracic spine			
Lumbar spine			
Elbow			
Hand and/or wrist			
Biceps			
Triceps			
Pectoral muscles			
Ab muscles			
Hip/groin			
Gluteal muscles			
Thigh muscles			
Knee			

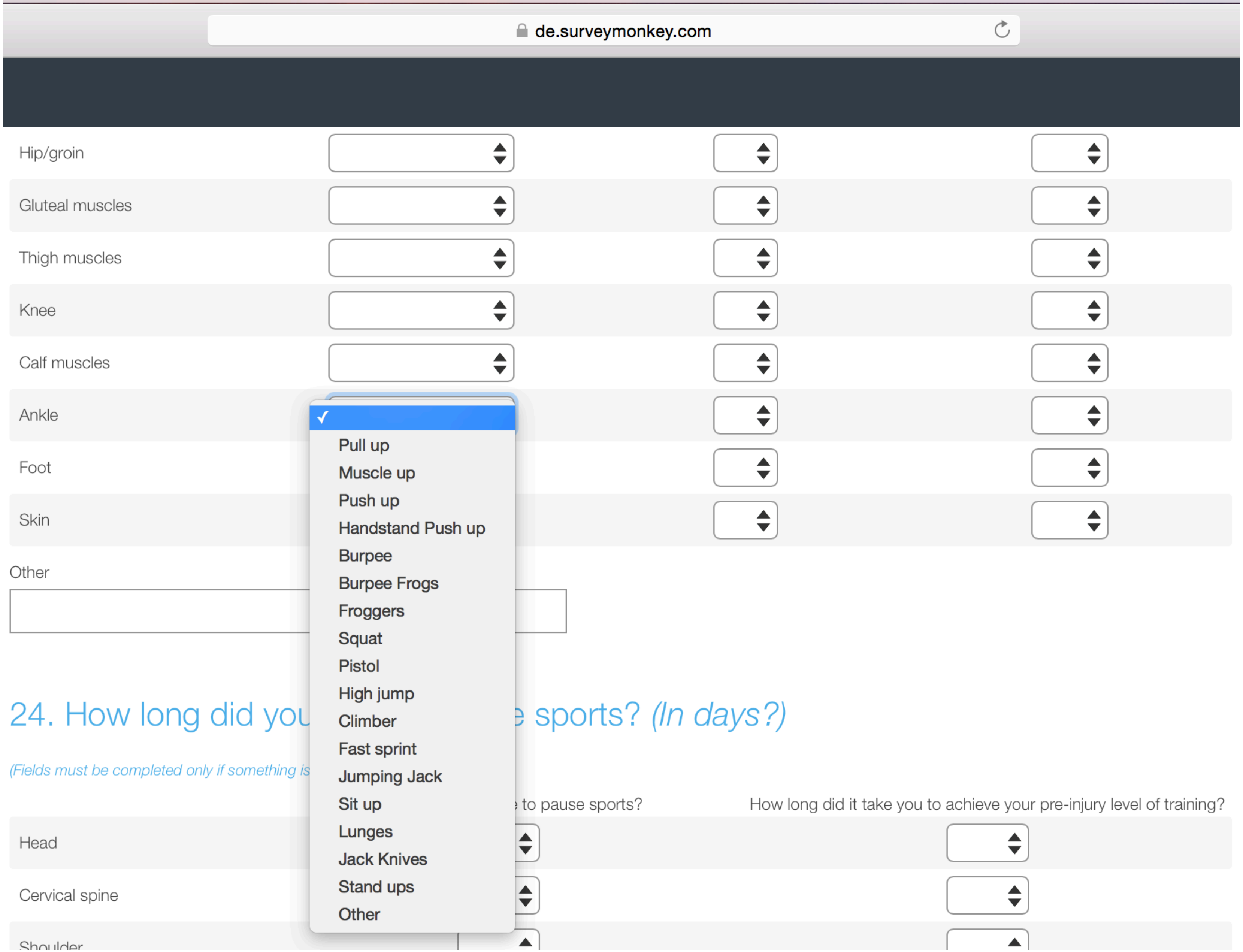
de.surveymonkey.com
Hip/groin
Gluteal muscles
Thigh muscles
Knee
Calf muscles
Ankle
Foot
Skin
Other
Pull up
Muscle up
Push up
Handstand Push up
Burpee
Burpee Frogs
Froggers
Squat
Pistol
High jump
Climber
Fast sprint
Jumping Jack
Sit up
Lunges
Jack Knives
Stand ups
Other
24. How long did you
sports? (In days?)
(Fields must be completed only if something is
to pause sports?
How long did it take you to achieve your pre-injury level of training?
Head
Cervical spine

de.surveymonkey.com

24. How long did you have to pause sports? *(In days?)*

(Fields must be completed only if something is correct!)

	How long did you have to pause sports?	How long did it take you to achieve your pre-injury level of training?
Head		
Cervical spine		
Shoulder		
Thoracic spine		
Lumbar spine		
Elbow		
Hand and/or wrist		
Biceps		
Triceps		
Pectoral muscles		
Ab muscles		
Hip/groin		
Gluteal muscles		
Thigh muscles		
Knee		

de.surveymonkey.com

Calf muscles		
Ankle		
Foot		
Skin		

Other

de.surveymonkey.com

25. What do you think caused the injury?

(Fields must only be filled in if something applies)

	What do you think caused the injury?
Head	
Cervical spine	
Shoulder	
Thoracic spine	
Lumbar spine	
Elbow	
Hand and/or wrist	
Biceps	
Triceps	
Pectoral muscles	
Ab muscles	
Hip/groin	
Gluteal muscles	
Thigh muscles	

Biceps	
Triceps	
Pectoral muscles	
Ab muscles	
Hip/groin	✓<br>Un-precise exercise execution<br>Too many repeats<br>Exhaustion<br>Pre-existing condition<br>Poor instructions<br>Other
Gluteal muscles	
Thigh muscles	
Knee	
Calf muscles	
Ankle	
Foot	
Skin	

Other

Zurück Weiter

de.surveymonkey.com

Freeletics Medical Survey English

26. Have you had problems with the injured body part **before** you started Freeletics?

(Fields must only be filled in if something applies!)

	Nature of the damage
Head	
Cervical spine	
Shoulder	
Thoracic spine	✓
Lumbar spine	
Elbow	
Hand and/or wrist	
Biceps	
Triceps	
Pectoral muscles	
Ab muscles	
Hip/groin	

Dropdown options:

- bruise
- fracture
- dislocation
- strain
- ligament strain
- tendinitis
- Periostitis
- Bone marrow
- muscle injury
- cartilage damage
- Abrasion / wound
- Something else

Pectoral muscles	
Ab muscles	
Hip/groin	
Gluteal muscles	
Thigh muscles	
Knee	
Calf muscles	
Ankle	
Foot	
Skin	

Etwas anderes

Zurück Weiter

Powered by
SurveyMonkey
Es ist ganz einfach, eine Umfrage zu erstellen.

de.surveymonkey.com

Freeletics Medical Survey English

27. How was the injury treated?

	Type of injury
No treatment	
Immobilization/rest	
Taping/splint	
Adapted training	
Pain medication	
Manual therapy (massage, ice)	
Rehabilitation exercises/stretching	
Acupuncture	✓
Other	

- bruise
- fracture
- dislocation
- strain
- ligament strain
- tendinitis
- Periostitis
- Bone marrow
- muscle injury
- cartilage damage
- Abrasion / wound
- Something else

Zurück

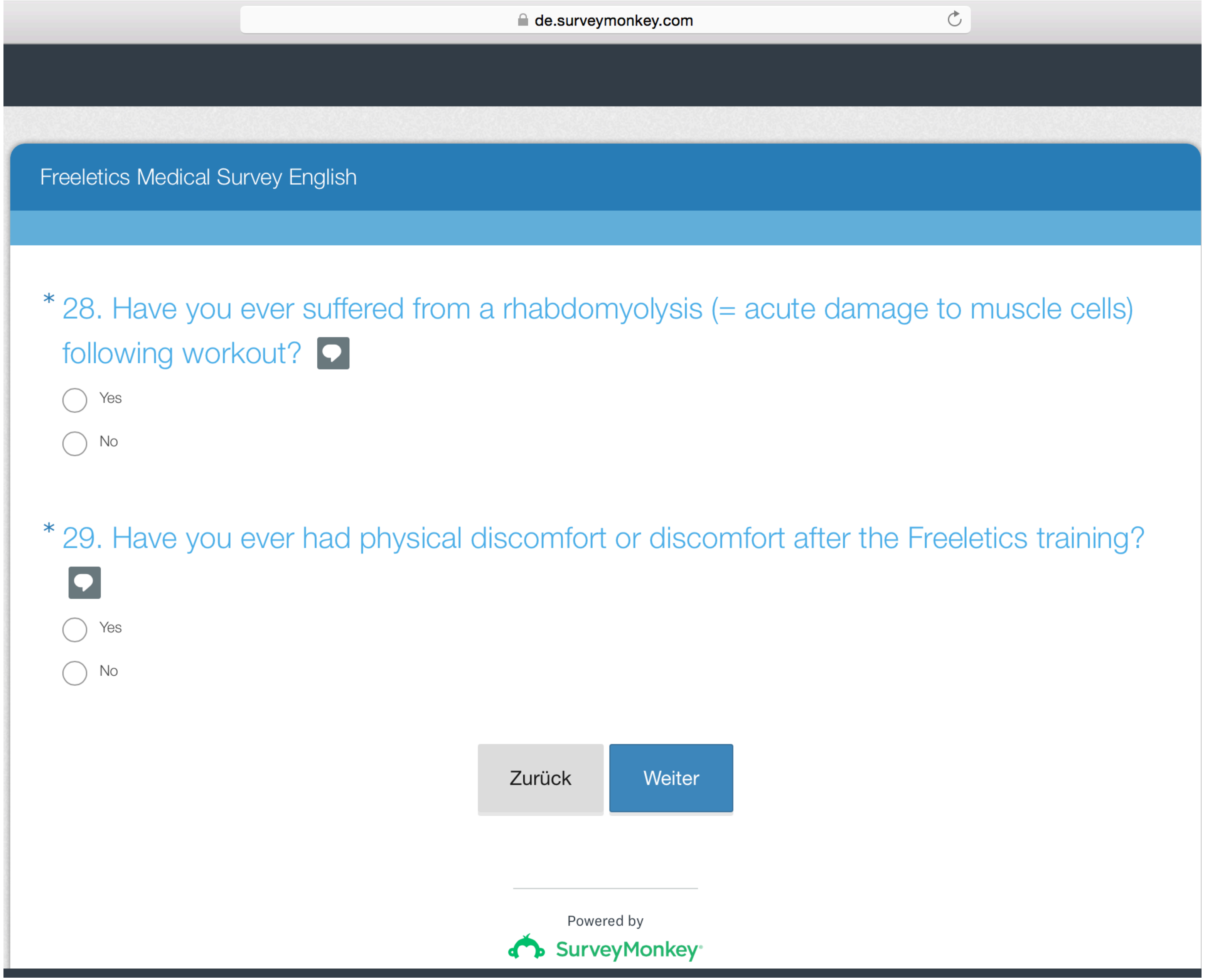
de.surveymonkey.com
Freeletics Medical Survey English
* 28. Have you ever suffered from a rhabdomyolysis (= acute damage to muscle cells) following workout?
Yes
No
* 29. Have you ever had physical discomfort or discomfort after the Freeletics training?
Yes
No
Zurück
Weiter
Powered by
SurveyMonkey

de.surveymonkey.com

Freeletics Medical Survey English

30. If so, which and in what percentage of the workouts such problems occurred?

	Percent
Severe exhaustion	
Muscle swelling	
Shortness of breath	
Nausea	
Tenderness of muscle to touch	
Muscle weakness	
Other	

✓
< 1%
1-5%
6-10%
11-20%
21-30%
31-40%
41-50%
51-60%
61-70%
71-80%
81-90%
91-100%

Zurück W

Powered by
SurveyMonkey
Es ist ganz einfach, eine Umfrage zu erstellen.

de.surveymonkey.com

Freeletics Medical Survey English

* 31. How exhausting do you perceive your average workout? (RPE scale)

- ◯ 0 = no exertion
- ◯ 1 = extemely light
- ◯ 2 = light
- ◯ 3 = moderate
- ◯ 4 = slightly exhausting
- ◯ 5 = exhausting
- ◯ 6 = difficult
- ◯ 7 = very difficult
- ◯ 8 = very very difficult
- ◯ 9 = barely feasible
- ◯ 10 = no longer feasible

de.surveymonkey.com

* 32. Have you always trained with the coach or have you freely chosen your workouts or supplemented by other sports?

(Multiple answer possible)

- [ ] Always trained with the coach
- [ ] Workouts freely combined in the app
- [ ] Combination of coach and additional workouts
- [ ] Combine with other sports
- [ ] When other sports, which?

* 33. What do you think could prevent problems or injuries in the future?

- [ ] Less repeats
- [ ] Slower training
- [ ] Better warm-up
- [ ] Better training technique
- [ ] Better training equipment
- [ ] Other training facility
- [ ] Other

de.surveymonkey.com

Freeletics Medical Survey English

* 34. I feel now, compared to before I started the Freeletics training (health)

- Much better than before
- Something better than before
- About the same
- Slightly worse than before
- Much worse than before

* 35. I feel now, compared to before I started the Freeletics training (psychological mood)

- Quieter and more relaxed than before
- somewhat more relaxed than before
- equal
- less relaxed than before
- inwardly distressed and agitated

de.surveymonkey.com

* 36. Through FL training, I can enjoy **everyday activities** such as shopping, strolling, cycling, carrying heavy objects **easier**:

- That is completely right
- Something is true
- Does not apply
- Not correct at all

* 37. Freeletics helped me get **better** in other sports:

- That is completely right
- Something is true
- Does not apply
- Not correct at all

Zurück Weiter

Powered by
SurveyMonkey
Es ist ganz einfach, eine Umfrage zu erstellen.

de.surveymonkey.com

[illegible]

* 38. I have found **friends** through the sport (in the community, for example) and made social contacts:

- That is completely right
- Something is true
- Does not apply
- Not correct at all

* 39. In everyday life with Freeletics training I feel

(only 1 answer possible)

- energetic
- concentrated
- more relaxed
- fortunate
- tired
- stressed out
- nothing like that
- Other

de.surveymonkey.com

* 40. In the future, I would like to (referred to my Freeletics Training)

(Multiple choice possible)

- [ ] train more
- [ ] exercise more regularly
- [ ] train less
- [ ] do more other sports
- [ ] stop with the FL training
- [ ] nothing like that
- [ ] Other

Zurück Weiter

Powered by
SurveyMonkey
Es ist ganz einfach, eine Umfrage zu erstellen.

de.surveymonkey.com

Freeletics Medical Survey English

Athlete,

it's almost done, there are only 2 questions left!

Clap Clap!

* 41. Would you rather see an orthopaedist who knows about Freeletics and is familiar with your sports?

- ◯ Yes
- ◯ No
- ◯ I don't care

* 42. Do you use other fitness apps besides Freeletics?

- ◯ Yes
- ◯ No
- ◯ If yes, which?
